# Effects of piezocision in orthodontic tooth movement: A systematic review of comparative studies

**DOI:** 10.4317/jced.56328

**Published:** 2019-11-01

**Authors:** Daniel-Santos-Fonseca Figueiredo, Ricardo-Gontijo Houara, Larissa-Salgado-da Mata-Cid Pinto, Amanda-Rafaela Diniz, Vânia-Eloisa de Araújo, Lehana Thabane, Rodrigo-Villamarim Soares, Dauro-Douglas Oliveira

**Affiliations:** 1 PhD, Pontifical Catholic University of Minas Gerais, Belo Horizonte, Brazil; 2 MSc in Dentistry, Pontifical Catholic University of Minas Gerais, Belo Horizonte, Brazil; 3 Dentist, Pontifical Catholic University of Minas Gerais, Belo Horizonte, Brazil; 4 Associate Professor, Pontifical Catholic University of Minas Gerais, Belo Horizonte, Brazil; 5 Department of Health Research Methods, Evidence, and Impact, McMaster University, Hamilton, Canada; 6 Associate Professor and Dean of Graduate Studies, Pontifical Catholic University of Minas Gerais, Belo Horizonte, Brazil; 7 Associate Professor and Program Director of Orthodontics, Pontifical Catholic University of Minas Gerais, Belo Horizonte, Brazil

## Abstract

**Background:**

The aim of this systematic review was to evaluate the effects of piezocision in accelerating orthodontic tooth movement (OTM) and its possible adverse effects.

**Material and Methods:**

The Databases Medline, Embase, CENTRAL and LILACS were searched until March 2019, for randomized controlled trials (RCTs) and controlled clinical trials (CCTs) that used piezocision associated with orthodontic treatment. A manual search was also performed. The search, studies selection, assessment of risk of bias and data collection were carried out by two independent reviewers.

**Results:**

Eleven publications were included in this review (4 CCTs and 7 RCTs). No study presented low risk of bias. Different types of tooth movement were evaluated: lower anterior alignment, en-masse retraction, overall orthodontic treatment and canine distalization. A total of 240 participants were analyzed in the included studies. Seven studies found significant acceleration in the piezocision group, while two studies found no differences. Adverse effects regarding patient’s satisfaction, pain perception, or worsening of periodontal parameters were not observed. There was no consensus concerning anchorage loss and root resorption.

**Conclusions:**

The literature does not provide high-quality evidence to confirm that Piezocision results in significant OTM acceleration. Therefore, high-quality RCTs should be conducted to allow reliable conclusions about the effects of piezocision in orthodontics.

** Key words:**Piezosurgery, tooth movement techniques, orthodontics.

## Introduction

The long treatment duration is a common complaint of a significant number of orthodontic patients. Therefore, several studies investigating different techniques aiming to reduce treatment time have been conducted (1-7). In this regard, surgical techniques generate trauma in the alveolar bone to alter the physiological response and cause a local transitory increase in bone metabolism and a decrease in its density (8). This biological response is known as the regional acceleratory phenomenon (RAP) and has been associated with the acceleration of orthodontic tooth movement (OTM) (7).

Particularly, alveolar corticotomies have received considerable attention (9,10). This technique consists in raising full-thickness buccal and lingual mucoperiosteal flaps and performing bone injuries utilizing surgical burs, often combined with bone grafting materials to enhance alveolar bone thickness (7,11). However, since alveolar corticotomies are considered an invasive technique, other alternatives to obtain RAP and consequent acceleration of OTM have been proposed (12-16).

In this regard, the use of a piezoelectric tip has been proposed as a substitute to surgical burs in an attempt to decrease the trauma, since it allows more accurate cuts, reducing the chances of developing osteonecrosis (12). Subsequently, techniques implementing decortication without raising mucoperiosteal flaps such as corticision, piezopuncture (13) and micro-osteoperforations (14) have also been described. However, piezocision is the minimaly invasive surgical technique that has gained more prominence in the literature. In this procedure, short incisions are performed in the soft tissue to allow access of the piezoelectric tip to the cortical bone in the interradicular regions. Despite being considered minimally invasive, this technique allows the addition of bone or soft tissue grafts to correct gingival recessions or bone deficiencies (15,16).

The number of studies investigating piezocision effects in OTM has increased in recent years (14-20). However, there is no consensus in the existing clinical studies about its actual effectiveness, as well as in regard to the occurrence of adverse effects. Therefore, a detailed analysis of controlled clinical trials (CCTs) and randomized controlled trials (RCTs) investigating the use of piezocision associated to orthodontic treatment, by means of a systematic review, could help orthodontists and their patients to achieve more rational and scientific-based treatment decisions.

The aim of the present systematic review was to evaluate the effects of piezocision in accelerating OTM and to assess secondary effects on anchorage loss, periodontal parameters, root resorption, patient satisfaction and pain perception.

## Material and Methods

The present systematic review was carried out using the Preferred Reporting Items for Systematic Reviews and Meta-Analysis: the PRISMA statement.

-Protocol and Registration

The protocol was registered on the PROPERO National Institute of Health Research Database (www.crd.york.ac.uk/prospero, protocol CRD42017070038).

-Eligibility Criteria

The following selection criteria were applied:

1. Study design: controlled clinical trials (CCTs) and randomized controlled trials (RCTs), including split-mouth design;

2. Participants: patients of both genders and at any age, presenting good health and indication of orthodontic treatment;

3. Intervention: piezocision. The control group should have received only conventional orthodontic intervention associated or not with other type of technique to accelerate OTM;

4. Exclusion criteria: piezocision with raising of a mucoperiosteal flap;

5. Outcomes analysis: primary outcomes included measurements of OTM acceleration, such as the rate of tooth movement, the accumulative moved distance, and the total orthodontic treatment duration. Secondary outcomes were amount of anchorage loss, changes in periodontal parameters, development of root resorption, patient satisfaction or pain perception and other reported outcomes.

-Information Sources, Search Strategy, And Study Selection

An extended search with indexed terms and synonyms was performed in the following databases: Medline/PubMed (Medical Literature Analysis and Retrieve System Online), EMBASE, Cochrane Central Register of Controlled Trials (CENTRAL) and Latin American and Caribbean Health Sciences (LILACS), from inception until March 30, 2019. The unpublished literature was searched using ClinicalTrials.gov (www.clinicaltrials.gov). Furthermore, academic papers were searched in Open Thesis (www.openthesis.org), in the Catalog of Theses and Dissertations of CAPES (catalogodeteses.capes.gov.br) and in the Portal of the Digital Library of Theses and Dissertations of USP (www.teses.usp.br). Authors were contacted to identify unpublished trials and to clarify doubts when necessary. Manual searches in the main orthodontic periodicals were also implemented. Bibliographic references of included studies and of systematic reviews were verified. Studies published in English, Spanish or Portuguese were retrieved since the researchers were fluent on those languages. No restrictions were applied to date of publication.

Two independent reviewers carried out the evaluation of the studies for inclusion in the review, assessment of bias risk and data collection. In cases of disagreement, a third evaluator was recruited to obtain consensus.

-Data Items and Collection

Several data, such as study design, sample characteristics, country of origin, comparison groups, description of the surgical protocol, details of orthodontic intervention, OTM measurements, follow-up time and presence of conflicts of interest were also collected. Primary and secondary outcomes were also properly organized into tables.

-Risk of Bias across studies and quality of evidence (GRADE)

Bias risk assessment (high, unclear or low) was performed for RCT studies using the Cochrane Collaboration risk of bias tool. Seven criteria were evaluated in each study, including allocation sequence generation, allocation concealment, blinding of participants and personnel, blinding of assessors, incomplete outcome data, selective reporting of outcomes, and other bias. Studies showing low risk in all criteria were classified as having low risk of bias. Studies that presented an unclear risk of bias in any of the criteria were classified as having uncertain risk of bias. Studies that showed high risk in any of the criteria, were classified as having high risk of bias.

For non-randomized studies, the “Risk Of Bias In Non-randomized Studies - of Interventions” (ROBINS-I) was used to assess the risk of bias (low, moderate, serious or critical) (21). It included risk of bias due to confounding factors, selection of participants, classification of interventions, deviations from intended intervention, missing data, measurement of outcomes, and selection of the reported results. The overall risk of bias for each study was equal to the most severe level of bias found in any domain.

In addition, to rate the quality of evidence and strength of recommendations the Grading of Recommendations Assessment, Development and Evaluation (GRADE)(22) was used for each outcome: rate of tooth movement, anchorage loss, periodontal parameters, root resorption and patient perception.

-Summary Measures an Approach to Synthesis

Quantitative data synthesis (meta-analysis) was not performed due to the dissimilarities of the studies. Therefore, qualitative data analysis was implemented.

## Results

-Study Selection and Characteristics

**Figure 1:**
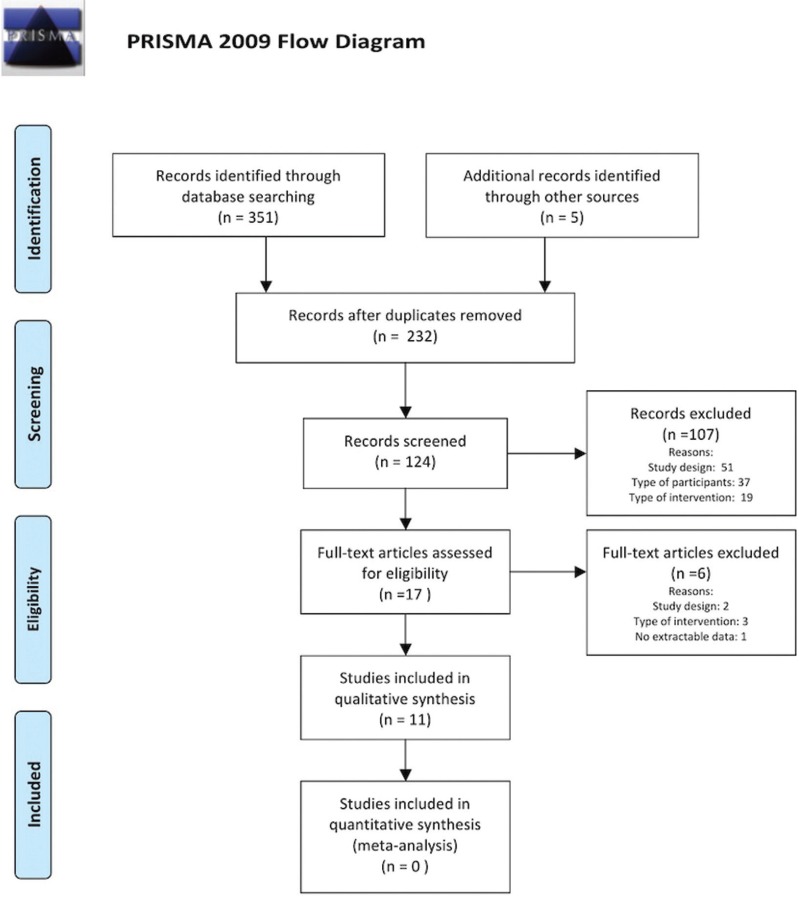
PRISMA flow diagram of the literature selection process.

Searches in the databases retrieved 351 publications. Using the manual search, 5 additional studies were found. With the removal of duplicates, a total of 232 publications were obtained. After reading the titles and abstracts and applying the eligibility criteria, 17 manuscripts were selected for complete reading. Six were removed due to study design (23,24), type of intervention (25-27) or absence of extractable data (28). At the end, 11 publications were left for qualitative analysis (Fig. 1).

Among all publications included in this study, seven were classified as RCT with parallel groups (18-20,29-32) and four as CCT (14,17,33,34). Although most of these studies have the same primary objective, significant differences regarding the type of orthodontic movement evaluated such as lower anterior alignment (20,29,31), en-masse retraction (19), canine retraction (30,14,17) and complete orthodontic treatment were observed (18,32,33). Comparison of piezocision with conventional alveolar corticotomies, laser-assisted flapless corticotomy and discision were also observed (17,30,33). Most included studies carried out the follow-up until the end of the orthodontic movement studied (14,18-20,29-33), one (17) evaluated the movement for only 3 months and another for 4 weeks (34). Sample-size calculations were performed in all RCTs and in one CCT (33). The total number of participants analyzed in these studies comprised 240 patients. The studies details are summarized in  Table 1,  Table 1 continue and 2- Table 2 continue-2.

**Table 1:**
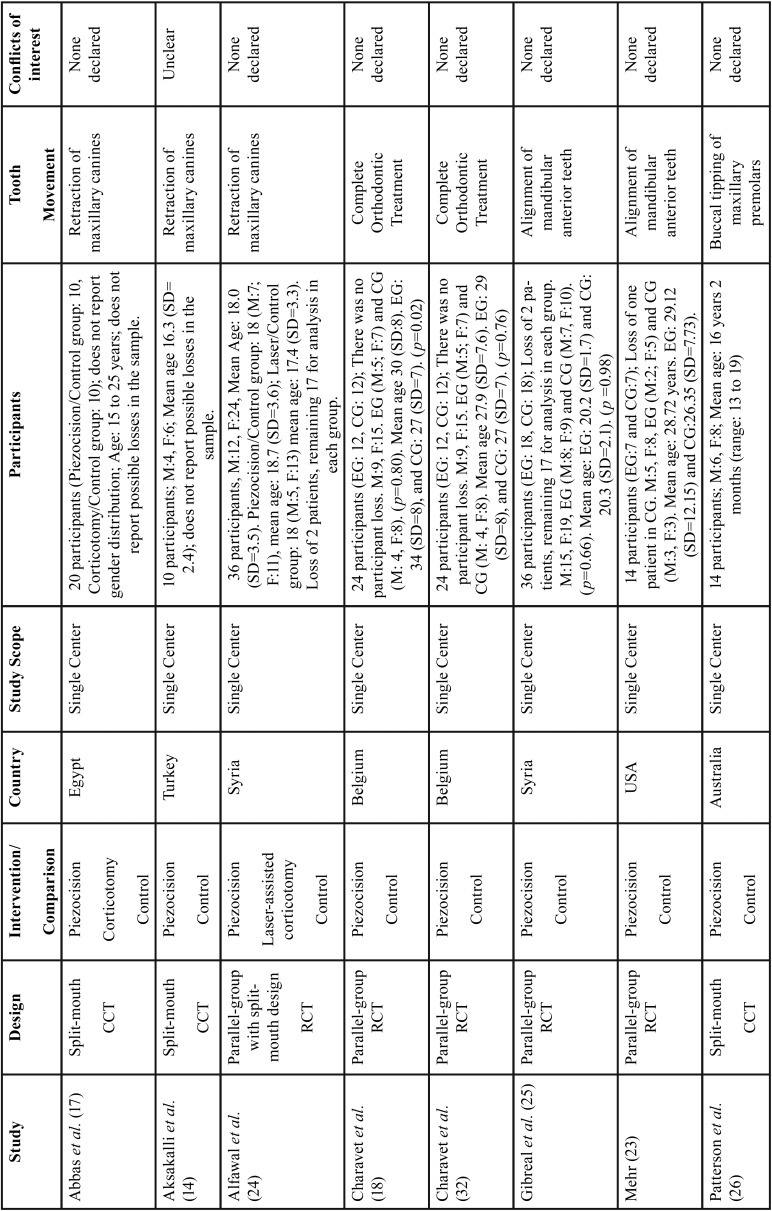
Characteristics of the included studies.

**Table 1 continue:**
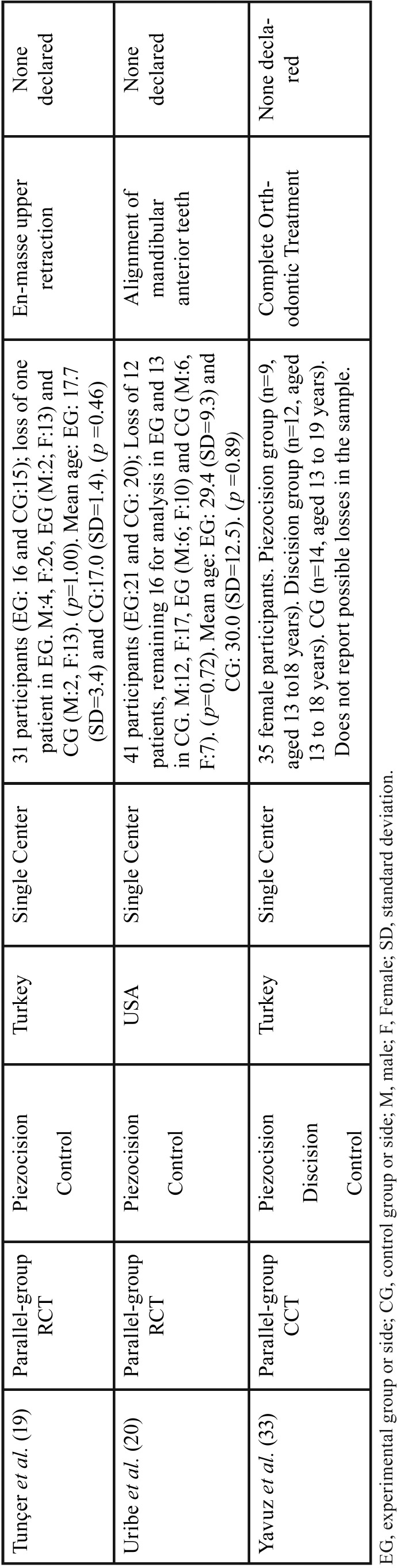
Characteristics of the included studies.

**Table 2:**
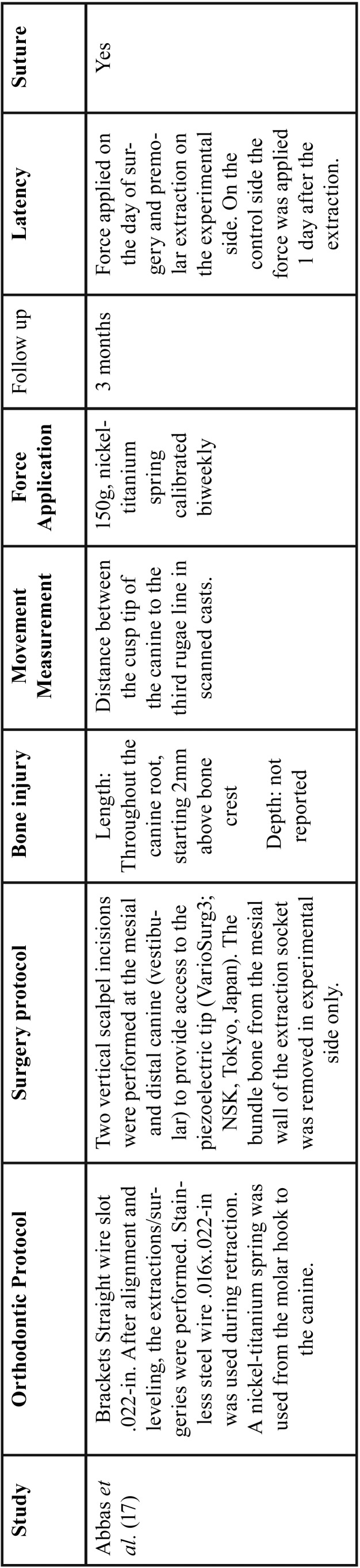
Extracted data of included studies.

**Table 2 continue:**
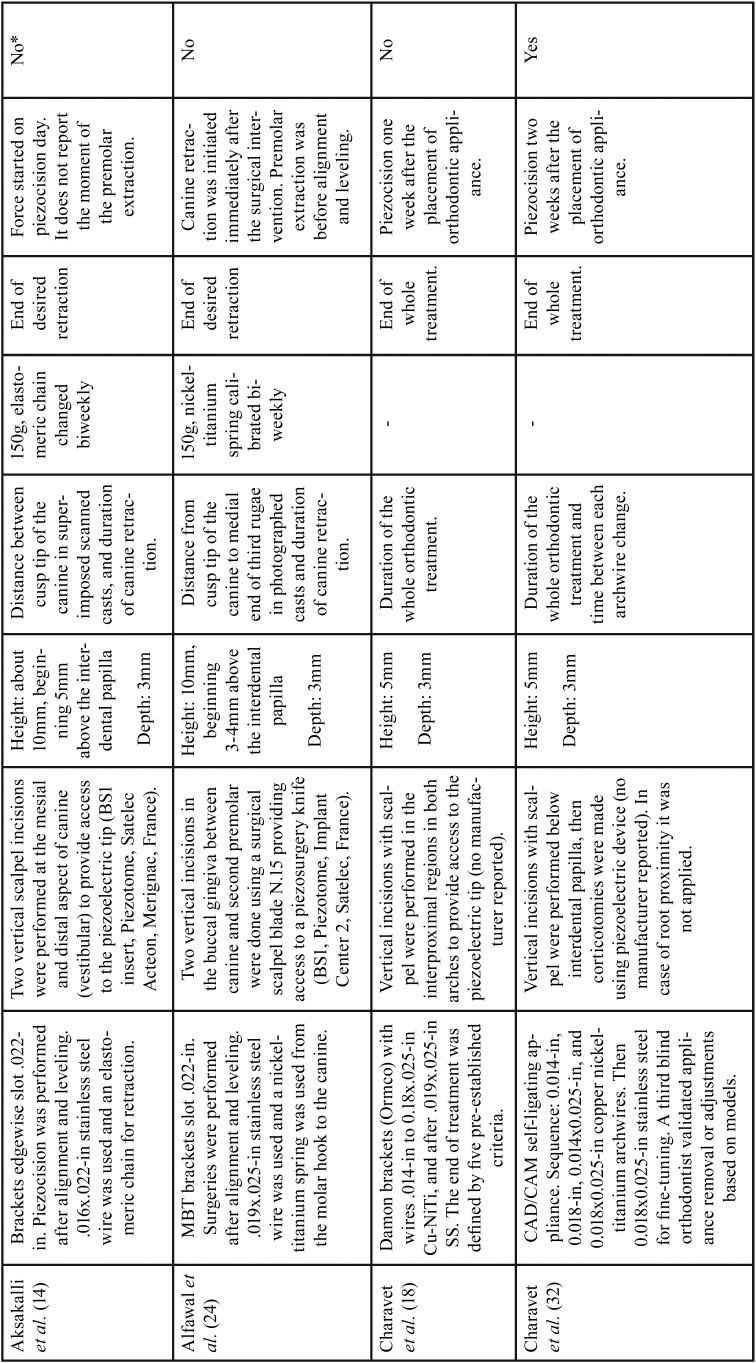
Extracted data of included studies.

**Table 2 continue-1:**
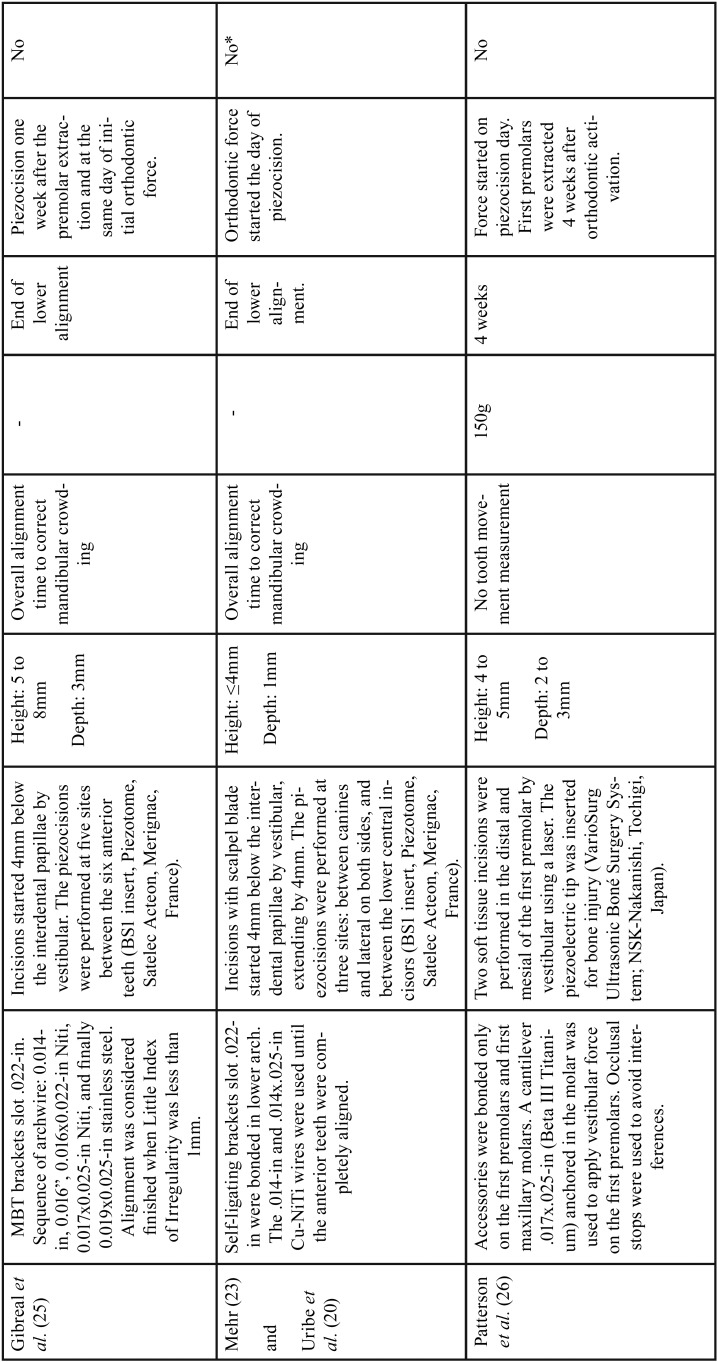
Extracted data of included studies.

**Table 2 continue-2:**
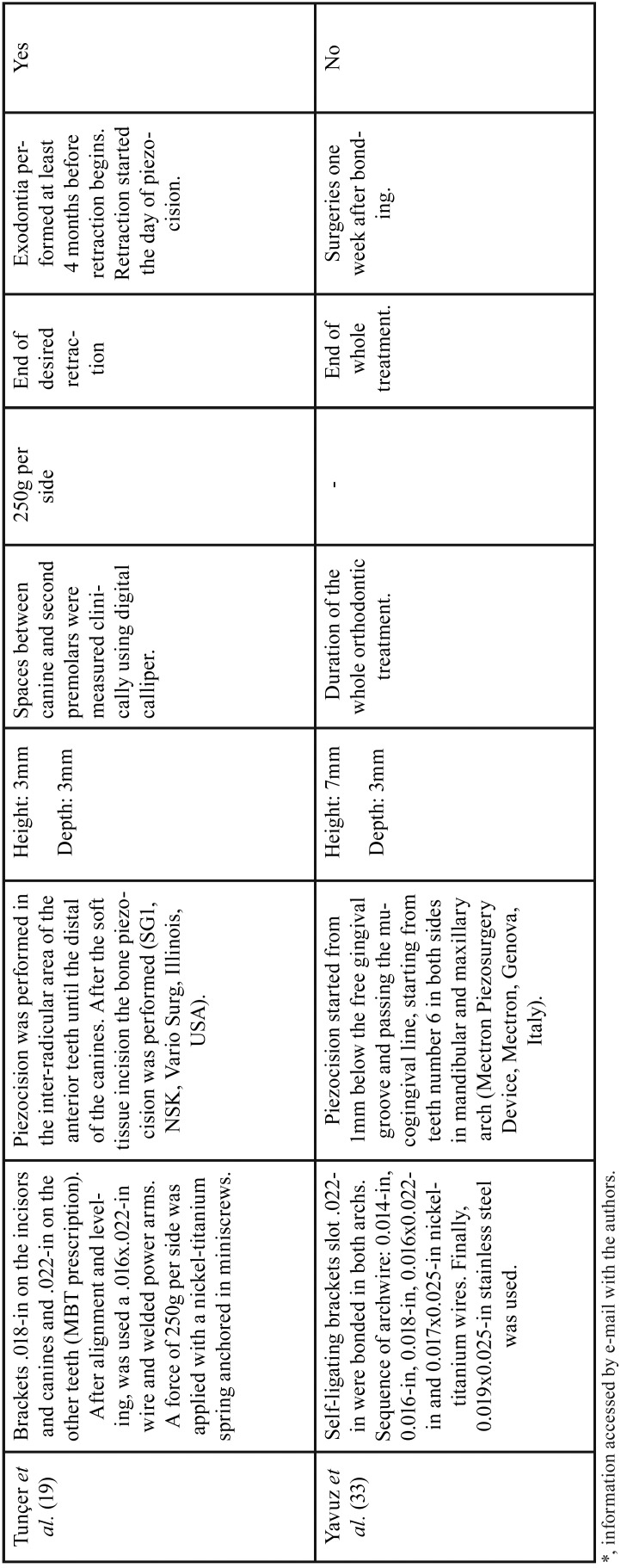
Extracted data of included studies.

-Risk of Bias Within Studies

**Figure 2:**
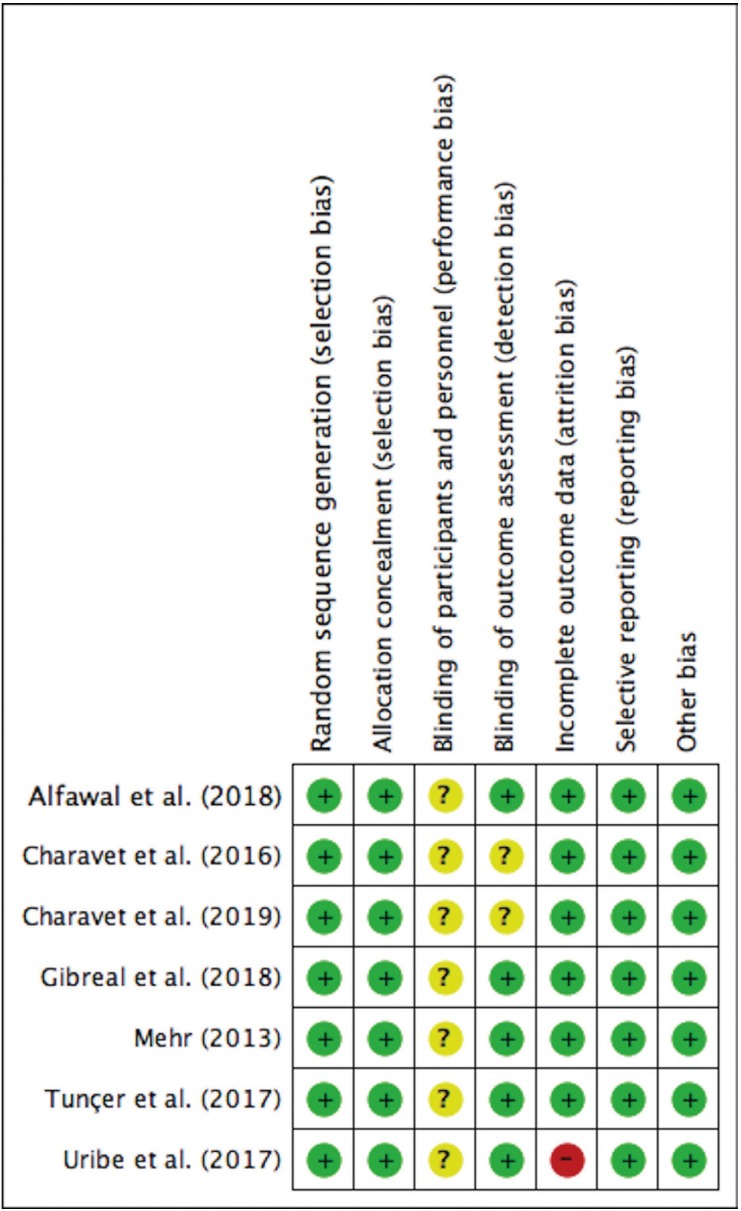
Risk of bias summary of RCT studies included.

Among the 7 RCTs evaluated, 6 presented an overall unclear risk of bias and 1 showed a high risk of bias (Fig. 2). Regarding selection bias, all RCTs were evaluated with low risk of bias since adequate random sequence generation and allocation concealment were observed.

Regarding performance bias, patient blinding was not possible in any of the studies due to the nature of the procedures. Blinding of personnel during the clinical follow-up of the patients was also not feasible in any of the studies mainly due to the presence of the soft tissue scars. Thus, to standardize the classification, this criterion was classified as having an unclear risk of bias.

Five RCTs showed low risk of bias detection since the evaluator did not have access to the side or group to be measured (19,20,29-31). Two studies did not describe this criterion with enough detail to allow a definitive judgment, and were classified as having an unclear risk (18,32). Regarding incomplete outcome data, only one study was classified as having high risk of bias due to a high attrition rate (20). Participants loss in the included RCTs did not occur or was considered small.

Among the four included CCTs, one was classified as having serious bias due to confounding, since the side of intervention was decided based in factors that predict the outcome of interest (32). The other CCTs were classified as having moderate risk of bias due to confounding factors, measurement of outcomes, and selection of the reported results (14,17,33) ( Table 3).

**Table 3:**
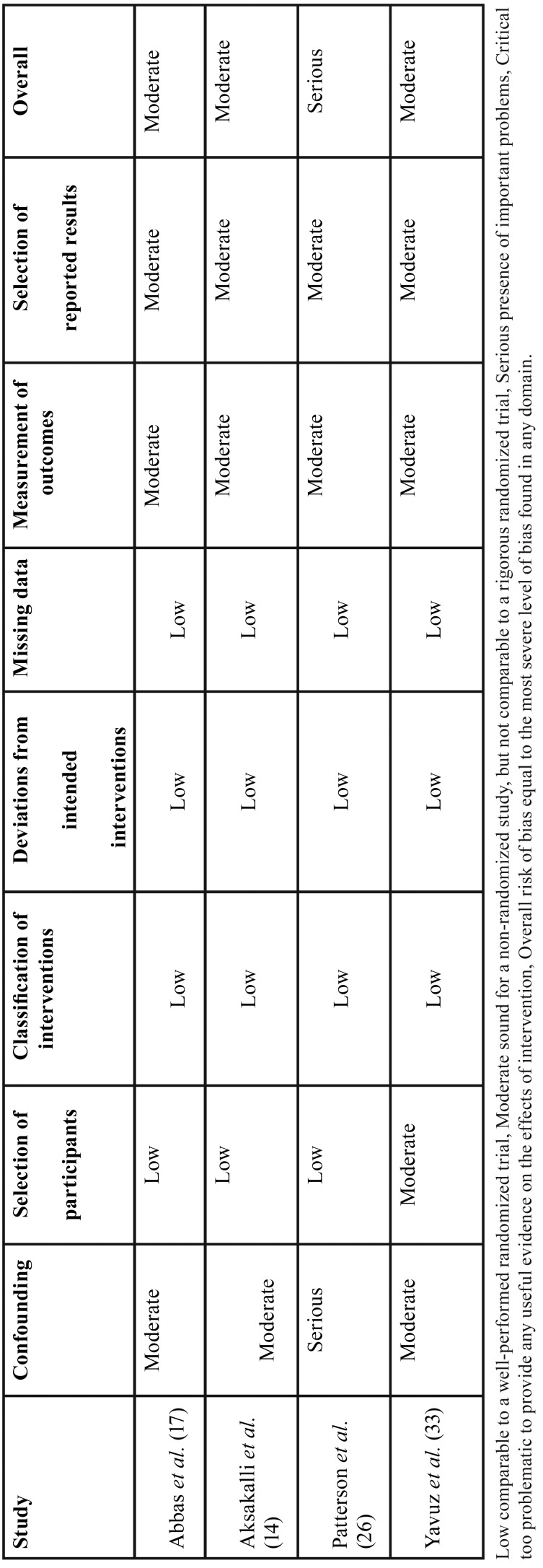
ROBINS-I (risk of bias judgements in non-randomized studies of interventions).

The quality of evidence for rate of tooth movement, anchorage loss and root resorption was considered low according to the GRADE system due to inconsistency and bias. The quality of evidence for periodontal parameters and patient perception (pain and satisfaction) was classified as moderate.

-Results of Specific Outcomes 

Rate of Tooth Movement 

The majority of the studies had OTM rate as the primary result (14,17-20,29-33). Abbas *et al.* (17) reported greater canine retraction rate in the piezocision side at all evaluated times (2, 4, 6, 8, 10 and 12 weeks), and also reported that the side submitted to corticotomies displayed greater rates of canine distalization than the side submitted to piezocision ( Table 4). Also evaluating canine distal movement, Aksakalli *et al.* (14) reported that a lower time period and a greater amount of retraction was observed on the piezocision side (3.54 ± 0.81 months of total time and 2.90 mm ± 0.86 of retraction in two months) in comparison to the control side (5.59 ± 0.94 months and 1.73 mm ± 0.72). Similarly, Alfawal *et al.* (30) reported that the piezocision side, in comparison to the control side, exhibited a two-fold greater canine retraction rate in the first month, and 1.5-fold in the second month (*p*<0.001), and an overall duration reduction of approximately 25% (*p*<0.001). Furthermore, the authors reported similar results when comparing piezocision and laser-assisted flapless corticotomy in canine distalization.30 In this context, Yavuz *et al.* (33) reported no significant difference in treatment time duration between piezocision and discision groups.

**Table 4:**
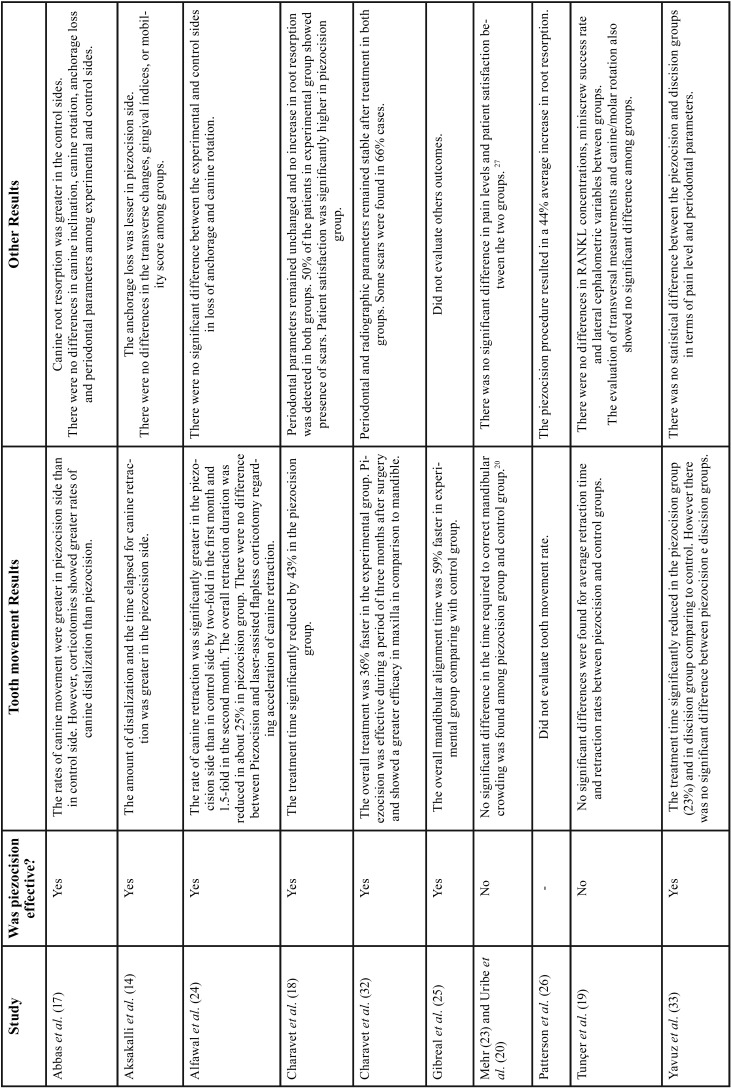
Outcomes of included studies.

In regard to the length of time necessary to correct the mandibular alignment, Uribe *et al.* (20) reported no difference (*p*= 0.52) between the piezocision (mean ± standard deviation (SD): 102.13 ± 34.73 days) and control groups (112 ± 46.2 days). In contrast, correcting severely crowded lower anterior teeth, Gibreal *et al.* (31) reported an overall alignment time 59% lower in the experimental group. Other studies evaluating the overall orthodontic treatment duration reported that the piezocision group exhibited a significant reduction (*p*<0.05) in mean treatment time than the control group (i.e.y 43%(18), 36%(32) and 23%(33)). Investigating the effect of piezocision on the en-masse retraction, Tunçer *et al.* (19) reported that although the piezocision group demonstrated a higher average amount of space closure on days 15, 30, 60, 90 and 120, the observed differences were not significant when compared to the control group results.

Anchorage Loss

Four studies evaluated anchorage loss. Abbas *et al.* (17) reported no difference between the loss of anchorage of the first molars between the experimental (3 mm ± 0.38) and control sides (3.25 mm ± 0.52) during canine retraction (*p*= 0.221). Similarly, Alfawal *et al.* (30) reported no difference in molar position between piezocision and control sides (*p*>0.05).(30) In contrast, Aksakalli *et al.* (14) reported greater anchorage loss of the first molars on the control side (3.01 mm ± 0.37) in relation to the piezocision side (2.04 mm ± 0.52). However, they only performed a descriptive analysis for this variable (14).

Periodontal Parameters

Five studies evaluated periodontal results (including probing depth, clinical attachment level, gingival recession, plaque and gingival index) after piezocision. (14,17,18,32,33) Significantly differences were not observed between piezocision and control groups for the periodontal parameters.

Root Resorption

Four studies evaluated root resorption after piezocision (17,18,32,34). In particular, this was the main objective of one study that applied orthodontic force to premolars during 28 days and subsequently conducted microcomputed tomographic analysis (34). The maxillary first premolars of the piezocision side showed significant (*p*= 0.029) higher root resorption (0.133 mm3) than the control side. In contrast, another study, that used cone-beam computed tomography, detected significantly (*p*<0.05) greater root resorption in the canines retracted on the control side in comparison to canines of the experimental side (submitted to piezocision or alveolar corticotomies) (17). A third study that measured root length before and after orthodontic treatment using computed tomographic scans, reported no difference between groups that received piezocision or were only submitted to orthodontic treatment (*p*> 0.05). (18) Similarly, the study conducted by Charavet *et al.* (32) reported no difference in root resorption between groups (*p*>0.05).

Patient Perception

Three studies assessed patient’s pain and discomfort perceptions using a visual analogue scale (VAS) (18,29,33). Two of them also evaluated patient’s satisfaction after treatment using questionnaires (18,29). One study described no significant differences in the pain level between the piezocision and control groups immediately, 1 hour, 12 hours, and 7 days after the first wire placement and activation (29). Moreover, the analgesic consumption was similar in both groups (29). Charavet *et al.* (18) used a VAS (ranging from 0 to 10) and reported that the mean pain level after piezocision was 6.0 ± 1.9, but they did not mention perception value observed in the control group. Yavuz *et al.* (33), compared VAS results between piezocision and discision group and reported no significant difference in pain perception among these surgical techniques.

In regard to patient satisfaction, similar levels of satisfaction (*p*> 0.05) of interest to undergo treatment again (84 a 86%) and to recommend the procedure to a friend in both groups were described in one study (29). However, another study reported that the levels of satisfaction were significantly higher in the piezocision group (*p*= 0.012) when compared to the control group (18). Moreover, in the piezocision group, a significantly greater numbers of patients reported that they would undergo the treatment again (*p*= 0.0009) and that they would recommend it to a friend (*p*= 0.0022) (18).

Other Outcomes

In the studies that evaluated canine retraction, no significant differences were found in canine tipping (17) and rotation, (17,30) the transversal dimension (14) or in tooth mobility (14), when control and piezocision sides were compared. During en-masse retraction, no difference was detected between groups for canine or molar rotation, as well as for the transversal dimension (19).

The success rate of miniscrews was also investigated in one study (19). In the group that received the piezocision, the rate was 86.7% and in the control group, it was 90%, a difference that was not statistically significant (19). The concentration of RANKL present in the gingival crevicular fluid was also compared and again, no significant differences between the groups were detected (19). Scars were reported in 50% (18) and 66% (32) of the participants submitted to piezocision.

## Discussion

-Summary of findings

Nine studies included in this review evaluated the effectiveness of piezocision in accelerating OTM (14,17-20,30-33) Significant acceleration in the experimental group was reported in 7 studies ( Table 4) (14,17,18,30-33). Differences in the surgical protocols performed, as well as from variations on the orthodontic treatment implemented could in part explain the observed differences. In thi regard, the studies that found OTM acceleration in the experimental group usually performed greater amounts of bone injury. It is recognized that RAP intensity and duration is proportional to the quantity of bone injury (8).

Abbas *et al.* (17) performed a long incision parallel to the entire extension of the canine root. However, an important bias is present in this study since the authors removed the bundle bone from the mesial wall of the premolar extraction socket only in the experimental side. Therefore, the surgical trauma was increased and bone resistance was reduced in the direction of the desired tooth movement (35). Therefore, the effect of piezocision in this study may be overestimated. The others split-mouth studies (14,30) that found significant acceleration in the experimental side, performed an extensive piezocision for canine retraction (10 mm), generating approximately twice the amount of bony injury described in the original technique (15). Evaluating lower anterior alignment in the orthodontic correction of severely crowded teeth, Gibreal *et al.* (31) reported significant acceleration after implementing significant injuries (five cortical incisions in the labial bone between the six anterior teeth with 5 to 8 mm in length and 3 mm in depth). With a parallel group design, Charavet *et al.*, (18,32) using piezocision in all interproximal spaces with a length of 5 mm and a depth of 3 mm, as well as Yavuz *et al.*, using 7mm in length and 3mm in depth (33), described acceleration in the total orthodontic treatment period.

The studies that did not report significant acceleration performed a lower amount of bone injuries. To evaluate the mandibular teeth alignment, Uribe *et al.* (20) performed only 3 incisions in the labial cortical plate, with 4mm of length and 1 mm of depth. Tunçer *et al.* (19) did not obseve significant acceleration after performing cuts of 3 mm length and 3 mm deepth to aid in upper en-masse retraction.

Four studies (17,18,32,34) evaluated root resorption after piezocision. In principle, the decrease in bone density would reduce a possible accumulation of excessive pressure in the periodontal ligament and subsequent occurrence of root resorption (34). However, consensus between the studies results did not occur. Therefore, additional investigations should be conducted to confirm this hypothesis.

Anchorage loss, as a secondary outcome, was reported in some studies (14,17,30). Two showed no difference between experimental and control groups (17,30). In contrast, one showed less anchorage loss in piezocision side (14). In principle, the transient RAP-induced osteopenia would decrease alveolar bone density nearby the tooth to be moved and therefore, require less effort of the non-corticotomized anchorage component and reduce its loss (11). However, there was no consensus to confirm this hypothesis for piezocision assisted OTM.

The studies that evaluated periodontal parameters derived from the surgical procedures did not report adverse effects (14,17,18,32,33). The presence of scars derived from the piezocision procedure was described in two studies (18,32). The authors recommended additional care when using this procedure in patients with a high smile line (18), even when sutures are implemented (32).

Two studies included in the present systematic review have an unique origin (20,29). It was chosen to include both studies since secondary outcomes, such as pain, discomfort and satisfaction with the treatment were not present in the later publication (20). However, the initial data related to OTM rate (29) was not considered in this review, and only the final data (20) was analyzed to avoid duplicated results.

-Limitations

The impact of bias on the outcome of systematic reviews is of great importance. None of the studies showed low risk of bias. According to the GRADE system, for the primary outcome, the quality of evidence was considered low due to bias and imprecision. Therefore, the results of this systematic review should be interpreted with caution. Publication bias is also an important issue due to positive results reports that could influence the external validity of this systematic review. (9)

The heterogeneity of the studies mainly regarding the type of orthodontic movement used and the adopted surgical protocol did not allow a balanced comparison between the results of the included studies and therefore, did not allow the performance of a meta-analysis.

## Conclusions

Although the majority of the included studies reported a tendency of OTM acceleration using piezocision, the quality of evidence is low to confirm that performing piezocision significantly accelerate orthodontic tooth movement. There is a need for well-conducted research with less risk of bias to allow solid conclusions in regard to the use of piezocision associated to orthodontic treatment.
